# Intermittent pneumatic compression for prevention of pulmonary thromboembolism after gynecologic surgery

**DOI:** 10.1186/1477-9560-3-18

**Published:** 2005-11-19

**Authors:** Nao Suzuki, Fumio Kataoka, Atsushi Higashiguchi, Takeshi Hirao, Sachiko Ezawa, Hiroyuki Nomura, Akiyo Tomita, Nobuyuki Susumu, Daisuke Aoki

**Affiliations:** 1 Department of Obstetrics and Gynecology, St. Marianna University School of Medicine, 2-16-1 Sugao, Miyamae-ku, Kawasaki-City, Kanagawa 216-8511, Japan; 2 Department of Obstetrics and Gynecology, School of Medicine, Keio University, 35 Shinanomachi, Shinjuku-ku, Tokyo 160-8582, Japan

## Abstract

**Background:**

To investigate the incidence of pulmonary embolism and risk factors for this condition after obstetric and gynecologic surgery, as well as the efficacy of intermittent pneumatic compression.

**Methods:**

A total of 6,218 patients operated at Keio University Hospital excluding obstetric or infertility-related surgery and uterine cervical conization were evaluated retrospectively to determine the preventive effect of intermittent pneumatic compression on postoperative pulmonary embolism.

**Results:**

Pulmonary embolism occurred in 42 patients (0.68%). Multivariate analysis showed that malignancy, blood transfusion, and a body mass index ≥25 kg/m^2 ^or ≥28 kg/m^2 ^were independent risk factors for postoperative pulmonary embolism. A significantly lower incidence of pulmonary embolism occurred in patients receiving pneumatic compression postoperatively versus those without it. Among gynecologic malignancies, endometrial cancer was a significant risk factor for pulmonary embolism.

**Conclusion:**

Preventive measures, including intermittent pneumatic compression, should be taken to avoid postoperative pulmonary thromboembolism in the gynecology field.

## Background

Previously, postoperative venous thromboembolism (VTE) did not attract much attention in Japan because its incidence was lower than in the USA and Europe [1]. However, an increasing number of patients have recently been diagnosed with VTE in Japan along with improved detection thanks to progress in imaging technologies and increasing medical interest in VTE. VTE is associated with pulmonary thromboembolism (PTE), which causes death in nearly 50% of patients if untreated and which appears to be caused by embolism arising from deep venous thrombosis. According to the statistics compiled by the Japanese Ministry of Health, Labor and Welfare, the number of deaths due to PTE increased more than 10-fold from 1951 to 2000 [2]. However, a recent report estimated the annual number of PTE patients in Japan at 3,492, which is approximately 1/25 of the number in the USA [3]. Prevention of VTE has been studied intensively in the USA and Europe since the American College of Chest Physicians (ACCP) Consensus Conference was held in 1985. Development of guidelines for the prevention of VTE has been discussed based on high-level evidence, and the seventh ACCP Consensus Statement [4] and the International Consensus Statement [5] were published in 2001. Although the incidence of VTE has been rapidly increasing in Japan, there is still a large difference from the incidence in the USA and Europe. Many deaths due to VTE with PTE may be regarded as sudden deaths because clinical diagnosis is difficult; therefore suitable Japanese guidelines for the prevention of VTE were compiled in 2004 [6].

The Japanese Society of Anesthesiologist (JSA) conducted a survey of perioperative PTE in 2003 and reported that there were 4.41 events per 10,000 operations; the number of PTE patients was third highest in the gynecologic field after the orthopedic and gastrointestinal surgery fields [7]. The incidence of VTE is higher in pregnant women than in non-pregnant women because of hypercoagulability, hypofibrinolysis, platelet activation, venous smooth muscle relaxation by female hormones, and venous compression by the enlarged uterus.

There have been few reports on postoperative PTE in the gynecologic field, apart from that by Nicolaids *et al*. [8], which indicated that the incidence of PTE was 40–80% higher after extended surgery for malignancy. Therefore, more data about VTE after gynecologic surgery, including that for malignant tumors, are needed. In the present study, we retrospectively investigated the incidence and risk factors for PTE after obstetric and gynecologic surgery performed at Keio University Hospital, and also evaluated the usefulness of intermittent pneumatic compression (IPC) for prevention of postoperative PTE.

## Methods

A total of 6,218 patients who underwent operations at the Department of Obstetrics and Gynecology, Keio University School of Medicine between January 1995 and December 2003 were analyzed in this study; operations excluded obstetric, infertility-related and uterine cervical conization surgeries. 42 patients were found to have developed postoperative PTE. As a control group, a total of 929 patients who underwent obstetric, infertility-related surgery and uterine cervical conization operations who did not develop PTE in either 1995 (471 patients) or 2002 (458 patients) were selected.

Univariate analysis was used to assess the relationship between PTE and risk factors, such as the age, body mass index (BMI), smoking habits, presence/absence of complications (hypertension, abnormal glucose tolerance, heart disease, and collagen disease), operating time, perioperative bleeding, surgical indication (benign or malignant disease), presence/absence of retroperitoneal lymph node dissection, and perioperative blood transfusion. We also investigated the relationship between PTE and risk factors, including the use of IPC, by multivariate logistic regression analysis. The patient pools were analyzed according to the following risk subcategories: age was divided into less than 40 years of age, between 40 and 50 years of age, and greater than 50 years of age; BMI was divided into less than 25 kg/m^2^, between 25 and 28 kg/m^2^, and greater than 28 kg/m^2^; operating time was divided into less than 4 hours, between 4 to 6 hours, and greater than 6 hours; and perioperative blood loss was divided into less than 1000 mL, between 1000 and 2000 mL, and greater than 2000 mL. At our hospital, IPC has been used in patients undergoing gynecologic surgery to prevent postoperative PTE since 1999, before which elastic bandages or stockings were used (both methods were employed during the transition year of 1998).

Symptomatic PTE patients were further evaluated by electrocardiography, arterial blood gas analysis, chest X-ray examination, or echocardiography. A confirmative diagnosis was made by chest helical computed tomography scan and pulmonary ventilation-perfusion scintigraphy. Furthermore, asymptomatic patients presenting with only a decreased transcutaneous oxygen saturation (SpO_2_) were similarly evaluated for confirmative diagnosis.

## Results

### Incidence of PTE

The overall incidence of PTE was 0.68% (42/6,218). In patients undergoing surgery for benign diseases, excluding obstetrics and infertility-related surgery, the incidence was 0.32% (10/3,158), while the incidence was 2.21% (32/1,451) in patients undergoing surgery for malignancy apart from uterine cervical conization.

### Symptoms

Symptoms were present in 27 of the 42 patients who had postoperative PTE, with chest pain and dyspnea occurring in approximately half of them (Table 1). No symptoms were observed in 15 patients who were diagnosed by a decreased SpO_2_. PTE was diagnosed at a mean of 2.69 days after surgery.

**Table 1:** Symptoms of PTE

Symptom	No. of patients
Chest pain	16
Dyspnea	15
Tachycardia	3
Chest discomfort	2
Palpitations	1
Hypotension	1
Cyanosis	1
Chills	1
Vomiting	1
No symptoms	15

Note: There were 42 patients, but some had multiple symptoms.

PTE: pulmonary thromboembolism

### Risk factors (Figure 1)

**Figure 1:**
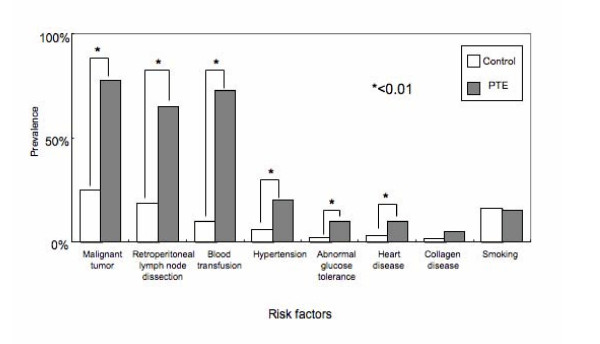
Prevalence of risk factors in patients with postoperative PTE. Six factors (malignancy, retroperitoneal lymph node dissection, blood transfusion, hypertension, abnormal glucose tolerance, and heart disease) were significantly associated with PTE.

We investigated the association between postoperative PTE and various risk factors, including the surgical indication (benign or malignant disease), perioperative blood transfusion, presence/absence of retroperitoneal lymph node dissection, smoking habits, and presence/absence of complications (hypertension, abnormal glucose tolerance, heart disease, and collagen disease). As a result, postoperative PTE was significantly associated with malignancy, perioperative blood transfusion, retroperitoneal lymph node dissection, hypertension, abnormal glucose tolerance, and heart disease.

### Influence of age and BMI

**Figure 2:**
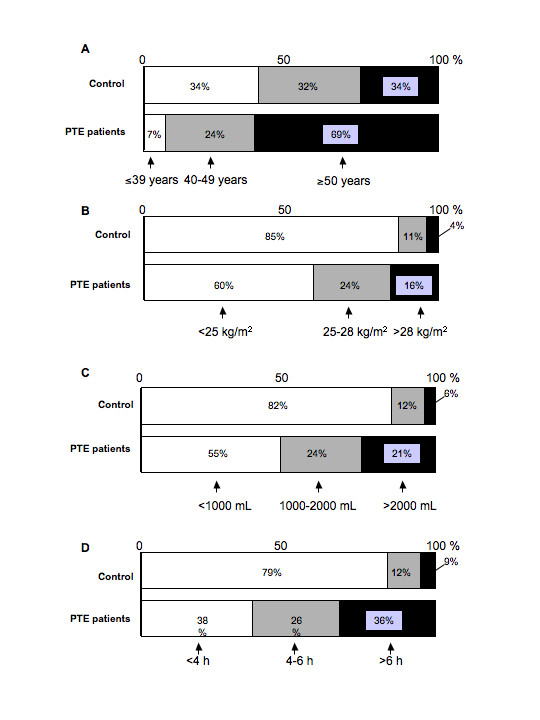
Distribution of risk factors in the postoperative PTE and control groups. A, age; B, BMI; C, perioperative bleeding; D, operating time.

Among the 42 patients with postoperative PTE, 93% were aged 40 years or older and 69% were aged 50 years or older (Figure 2-A). There was no such bias in the age distribution of the control group, with approximately 30% of the subjects in each age group. Patients with a BMI ≥25 kg/m^2 ^accounted for 40% of the PTE group versus 15% of the control group (Figure 2-B).

### Operating time and perioperative bleeding

Sixty-two percent of patients with postoperative PTE had an operating time of four hours or longer and 36% had a time of six hours or longer (Figure 2-C). In the control group, 21% of patients had an operating time of four hours or longer and 9% underwent operations lasting six hours or longer. In addition, perioperative bleeding was ≥1,000 mL in 45% of PTE patients and ≥2,000 mL in 21%. In the control group, however, perioperative bleeding was ≥1,000 mL in 18% of patients and ≥2,000 mL in 6% (Figure 2-D).

### Univariate analysis (Table 2)

**Table 2:** Results of univariate analysis

Factor	Odds ratio	p value (chi-square)
Age ≥40 years old	6.637	<0.01
Age ≥50 years old	4.362	<0.01
BMI ≥25 kg/m^2^	3.718	<0.01
BMI ≥28 kg/m^2^	3.911	<0.05
Smoking	0.809	n.s.
Hypertension	3.018	<0.01
Abnormal glucose tolerance	6.285	<0.05
Heart disease	2.951	<0.05
Collagen disease	2.161	n.s.
Malignant tumor surgery	8.327	<0.01
Retroperitoneal lymph node dissection	7.577	<0.01
Perioperative bleeding ≥1,000 mL	3.797	<0.01
Perioperative bleeding ≥2,000 mL	4.252	<0.01
Blood transfusion	7.152	<0.01
Operating time ≥4 hours	5.999	<0.01
Operating time ≥6 hours	5.516	<0.01

BMI: body mass index

n.s.: not significant

Analysis by the χ^2 ^test showed a significant association between PTE and seven background factors: age ≥40 years, age ≥50 years, BMI ≥25 km/m^2^, BMI ≥28 kg/m^2^, hypertension, abnormal glucose tolerance, and heart disease. A significant association was also observed with the following seven surgical factors: operation for malignancy, operating time ≥4 hours, operating time ≥6 hours, perioperative bleeding ≥1,000 mL, perioperative bleeding ≥2,000 mL, retroperitoneal lymph node dissection, and perioperative blood transfusion. These variables were also selected as risk factors.

### Multivariate analysis (Table 3)

**Table 3:** Results of multivariate analysis

Factor	Risk ratio	95% CI		p value (chi-square)
		Lower	Upper	
Age ≥40 years old	2.645	0.703	9.946	n.s.
Age ≥50 years old	3.418	0.926	12.615	n.s.
BMI ≥25 kg/m^2^	2.718	1.149	6.427	<0.05
BMI ≥28 kg/m^2^	3.922	1.297	11.863	<0.05
Smoking	1.029	0.364	2.913	n.s.
Hypertension	0.838	0.311	2.259	n.s.
Abnormal glucose tolerance	2.176	0.675	7.016	n.s.
Heart disease	1.149	0.304	4.345	n.s.
Collagen disease	1.181	0.199	7.018	n.s.
Malignant tumor surgery	2.860	1.083	7.522	<0.05
Blood transfusion	3.834	1.683	8.737	<0.01
IPC	0.396	0.193	0.814	<0.05

BMI: body mass index

CI: confidence interval

IPC: intermittent pneumatic compression

n.s.: not significant

Before performing multivariate analysis, the correlations between variables were investigated by calculating Spearman's correlation coefficients. There were strong correlations between malignancy, retroperitoneal lymph node dissection, operating time, and the amount of perioperative blood loss (data not shown), therefore we selected two of these factors related to surgery (malignancy and blood transfusion) for analysis.

As shown in Table 3, multivariate analysis was done for a total of 12 variables, including the use of IPC. Significant associations with PTE were observed in the case of surgery for malignant tumors, blood transfusion, BMI ≥25 km/m^2^, and BMI ≥28 kg/m^2^, with their risk ratios being 2.860, 3.834, 2.718, and 3.922, respectively. A significantly lower rate of PTE was observed in the patients treated with IPC (risk ratio of 0.396).

### Postoperative PTE and gynecologic diseases

Thirty-two patients among 42 postoperative patients who developed PTE had malignant tumors (76%) compared to an overall rate of PTE development of 2.21% (32/1,451) among all patients undergoing surgery for gynecologic malignancies.

There were 16 patients who had endometrial cancer among the 32 PTE patients with malignancy (Table 4). BMI was ≥25 kg/m^2 ^in 10/16 patients (62.5%) with endometrial cancer, 2/9 patients (22.2%) with ovarian cancer, and 2/6 patients (33.3%) with uterine cervical cancer (Table 5). There was also a significant difference of postoperative PTE between patients undergoing pelvic lymph node dissection alone and those undergoing combined pelvic and para-aortic lymph node dissection, with the incidence being 2.4% (15/636) and 6.2% (13/211), respectively (p < 0.01) (Table 6).

**Table 4:** Postoperative PTE and gynecologic diseases

Malignant tumors	No. of patients	Benign tumors	No. of patients
Endometrial cancer	16	Uterine myoma	3
Ovarian cancer	9	Uterine adenomyosis	3
Cervical cancer	6	Ovarian tumor	1
Others	1	Cystocele	1
		Uterine myoma/ovarian tumor	1
		Others	1
Total	32	Total	10

PTE: pulmonary thromboembolism

**Table 5:** Malignancy and BMI in patients with postoperative PTE

		Endometrial cancer	Ovarian cancer	Cervical cancer
BMI	<25 kg/m^2^	6	7	4
	25–27 kg/m^2^	7	0	2
	≥25 kg/m^2^	3	2	0
	Total	16	9	6

BMI: body mass index

PTE: pulmonary thromboembolism

**Table 6:** Postoperative PTE and lymph node dissection

Site	Incidence of postoperative PTE	
Pelvic lymph nodes	2.4% (15/636)	p < 0.01
+ Para-aortic lymph nodes	6.2% (13/211)	

PTE: pulmonary thromboembolism

On the other hand, 10 of the 42 patients (24%) who developed PTE had benign disease compared to an overall rate of PTE development of 0.32% (10/3,158) among all patients undergoing surgery for benign gynecologic tumors.

Patients with uterine myoma and uterine adenomyosis accounted for almost half of these 10 patients (Table 4).

### Effect of IPC

There was a significant difference between the incidence of postoperative PTE before and after the introduction of IPC, being 1.19% (23/1,928) versus 0.40% (14/3,525), respectively (p < 0.01) (Table 7).

**Table 7:** Prophylaxis for postoperative PTE

	1995–1997	1998	1999–2003
No prevention	9	0	0
Elastic bandages	4	1	0
Elastic stockings	10	4	0
IPC	0	0	14

Before vs. after introduction of IPC: 1.19% (23/1,928) vs. 0.40% (14/3,525) (p < 0.01)

IPC: intermittent pneumatic compression

PTE: pulmonary thromboembolism

## Discussion

Based upon a survey conducted by The Japan Society of Obstetrics, Gynecology, and Neonatal Hematology between 1991 and 2000 at 92 medical institutions in Japan, the incidence of postoperative PTE was 0.08% (168/203,058) in all patients undergoing gynecologic surgery, with a breakdown of 0.03% (51/175,448) in patients undergoing surgery for benign disease compared to 0.42% (117/27,610) in patients undergoing surgery for malignant disease.

The incidence was approximately 14 times higher in patients undergoing surgery for malignancy than in patients undergoing surgery for benign disease [9]. The present study retrospectively investigated the incidence of PTE after gynecologic surgery excluding obstetric surgery, infertility-related surgery, and uterine cervical conization, and we found that the incidence of postoperative PTE was 0.68% (42/6,218), which was higher than the average incidence in Japan. This result appears to be attributable to the high percentage of patients with malignancy among those undergoing gynecologic surgery in our hospital, since patients who had malignant disease surgery accounted for 76.5% of the 42 patients with postoperative PTE. The relationship between VTE and malignancy has long been known as Trousseau's syndrome. It has been reported that VTE is caused by release of procoagulant factors from cancer cells and direct damage to venous endothelial cells and that the incidence of PTE is 3–5 times higher in patients with malignancy [10]. In addition, cancer patients tend to be older and more often have complications, such as hypertension, abnormal glucose tolerance, and heart disease. Furthermore, surgery for malignancy requires a longer operating time, causes more bleeding, and often requires blood transfusion. These were all significant risk factors according to univariate analysis (χ^2 ^test) in the present study. In addition, multivariate analysis selected malignancy as an independent risk factor for postoperative PTE along with blood transfusion and BMI (≥25 kg/m^2 ^or ≥28 kg/m^2^). There have been few reports on VTE after gynecologic surgery; Horowitz found that obesity, a long period of immobilization, extensive cancer surgery, trauma, radiotherapy, a past history of VTE, severe varices, diabetes, and heart failure were risk factors of postoperative VTE [11]. This report is comparable with results of the present study. Our results are also comparable with the ACCP guidelines, which categorize patients ≥40 years old with extensive surgery and malignant tumors as the highest-risk group [4]. The JSA has also reported that obesity, long-term immobilization, and malignant tumors are risk factors for perioperative PTE, especially in female patients [7]. Of the 32 patients with postoperative PTE in the present study, 50% had endometrial cancer, which is often associated with obesity, hypertension, and abnormal glucose tolerance.

Endometrial cancer is increasing relative to cervical cancer in Japan as well as the USA and Europe. Therefore, endometrial cancer appears to be one of the strongest risk factors for postoperative PTE among malignant gynecologic tumors. Thrombosis occurs due to Virchow's triad, namely 1) hypercoagulability, 2) stagnation of blood, and 3) vascular endothelial cell damage. Retroperitoneal lymph node dissection was not identified as an independent risk factor for PTE according to multivariate analysis in the present study. Lymph node dissection may be closely related to the occurrence of VTE since this procedure causes vascular damage and also accumulations of lymph may compress the veins after surgery and cause stagnation of blood. As shown in Table 6, the incidence of postoperative PTE was increased in patients who had both pelvic and para-aortic lymph node dissection.

Retroperitoneal lymph node dissection is important for the treatment of endometrial cancer, but these findings suggest that it is necessary to carefully consider the performance of lymph node dissection in patients with a number of risk factors as well as the use of postoperative radiotherapy.

There are also patients with benign gynecologic disease in whom attention should be paid to the risk of postoperative PTE. Of the 10 patients with benign gynecologic disease in the present series, six were had relatively large uterine tumors, including myoma and adenomyosis. Although age is specified as a risk factor in the ACCP guidelines [4], neither an age ≥40 years nor an age ≥50 years was identified as a risk factor in the present study. However, multivariate analysis by the χ^2 ^test showed a significant difference for patients ≥50 years old (p = 0.0651) in this study, indicating that elderly patients are at increased risk of developing postoperative PTE.

As well as minimizing risk factors, it is important to employ adequate preventive measures for VTE in high-risk patients according to the guidelines. Multivariate analysis showed that the risk ratio of patients treated with IPC was 0.396 (p < 0.05), so this technique was an independent preventive factor for postoperative PTE. Our department introduced IPC in 1999, as did other institutions in Japan [6]. Comparison of the incidence of postoperative PTE before and after the introduction of IPC showed a significant decrease from 1.19% to 0.40%. In addition, the five patients experiencing postoperative PTE in 1998 (transitional period for introduction of IPC) were all managed with other preventive methods. The ACCP guidelines state that IPC reduces the risk of postoperative VTE by 88%, which is superior to the risk reduction rate for low-dose unfractionated heparin (68%) or low-molecular-weight heparin (76%), suggesting that IPC is one of the most useful preventive methods for VTE and PTE [5].

The JSA reported that the incidence of perioperative PTE is 4.41/10,000, with the mortality rate being 18% [7]. It also reported that 57.7% of PTE was likely to be preventable. In Japan, there is a need to accumulate more evidence-based clinical data, in order to better define the risk factors for VTE and PTE and allow the selection of appropriate preventive methods.

## Conclusion

Preventive measures, including intermittent pneumatic compression, should be taken to avoid postoperative pulmonary thromboembolism in the gynecology field.

## List of abbreviations

ACCP American College of Chest Physicians

BMI body mass index

IPC intermittent pneumatic compression

JSA Japanese Society of Anesthesiologist

PTE pulmonary thromboembolism

SpO_2 _oxygen saturation

VTE venous thromboembolism

## Competing interests

The author(s) declare that they have no competing interests.

## Authors' contributions

NS (Suzuki) and SE were involved in the sequence alignment and drafted the manuscript.

HN and NS (Suzuki) were involved in writing of method.

NS (Suzuki) and FK were involved in writing manuscript.

FK, AH, SE, and HN were involved in analysis of data.

*** was involved in the sequence alignment.

NS (Suzuki), FK, and DA were involved in the design of the study.

NH, AT and NS (Susumu) performed the statistical analysis.

AH and HT helped to draft the manuscript.

NS (Suzuki), NS (Susumu) and DA were involved in planning, experimental setup.

All authors read and approved the final manuscript.
